# Polyphenolic Characterization, Antioxidant, Antihyaluronidase and Antimicrobial Activity of Young Leaves and Stem Extracts from *Rubus caesius* L.

**DOI:** 10.3390/molecules27196181

**Published:** 2022-09-21

**Authors:** Anna Hering, Justyna Stefanowicz-Hajduk, Rafał Hałasa, Marta Olech, Renata Nowak, Piotr Kosiński, J. Renata Ochocka

**Affiliations:** 1 Department of Biology and Pharmaceutical Botany, Medical University of Gdańsk, 80-416 Gdańsk, Poland; 2 Department of Pharmaceutical Microbiology, Medical University of Gdańsk, 80-416 Gdańsk, Poland; 3 Department of Pharmaceutical Botany, Medical University of Lublin, 20-093 Lublin, Poland; 4 Faculty of Agriculture, Horticulture and Bioengineering, Poznań University of Life Sciences, 60-637 Poznań, Poland

**Keywords:** the European dewberry, antihyaluronidase, *antibacterial*, *Clostridium bifermentans*, *Clostridium sporogenes*, *Enterococus faecalis*, DPPH, FRAP, polyphenols, tiliroside, hyperoside

## Abstract

Fruits are the main food part of the European dewberry (*Rubus caesius* L.), known as a source of polyphenols and antioxidants, while very little attention is paid to leaves and stems, especially young first-year stems. The purpose of this work was to analyze for the first time water and ethanol extracts obtained from young, freshly developed, leaves and stems of the European dewberry to determine their antioxidant and biological activity, whereas most of the papers describe biological properties of leaves collected during summer or autumn. As the phytochemical profile changes during the growing season, the quantitative and qualitative content of flavonoid glycosides and flavonoid aglycones was analyzed using reversed phase liquid chromatography/electrospray ionization triple quadrupole mass spectrometry (LC-ESI-MS/MS) with multiple reaction monitoring (MRM). The ability to inhibit hyaluronidase as well as antioxidant activity (2,2 diphenyl-1-picrylhydrazyl: DPPH and ferric antioxidant power: FRAP) were estimated. Extracts were also analyzed against Gram-positive and Gram-negative bacteria. The results of the qualitative phytochemical analysis indicated the presence of flavonoid aglycones and flavonoid glycosides, with the highest amount of tiliroside, hyperoside, isoquercetin, astragalin, rutin and catechin in ethanol extracts. DPPH and FRAP tests proved the high antioxidant activity of the extracts from leaves or stems and the antihyaluronidase assay revealed for the first time that water and ethanol extracts obtained from the stems exhibited the ability to inhibit hyaluronidase activity resulting in an IC_50_ of 55.24 ± 3.21 and 68.7 ± 1.61 μg/mL, respectively. The antimicrobial activity has never been analyzed for European dewberry and was the highest for *Clostridium bifermentans* and *Clostridium sporogenes*—anaerobic sporulation rods as well as *Enterococcus faecalis* for both water and ethanol extracts.

## 1. Introduction

In urban society, diseases of civilization, such as diabetes, cancer, obesity, atherosclerosis, as well as allergies are observed more and more often [1]. Additionally, poor nutrition, rich in highly processed food, often aggravates health problems [2]. Natural products are nowadays in demand to improve and support the functioning of human organisms, especially those exposed to stress, tobacco smoke or industrial smog. Polyphenols are a large group of natural compounds widely spread among plants. They are well known for the ability to protect cells from free radicals–responsible for many diseases [3]. The use of polyphenol rich plants as a functional food might support the defense mechanisms of the organisms exposed to risk factors. According to the theory of “food as medicine” inflammation or disease processes may be reduced with nutraceutical ingredients introduced to the diet [4].

The European dewberry (*Rubus caesius* L., *Rosaceae*) is a widely distributed species in many parts of Europe and Asia. In Poland, it is one of the commonest brambles occurring along the roads and forest margins. This small shrub has trailing or low-arching shoots and pruinose aggregate fruit consisting of several drupes. The fruits are eaten directly from the plant or could be used to produce jams or cakes. It was reported that the fruits and the seeds contain significant amounts of flavonoids and anthocyanins [5,6,7]. The leaves and stems are also a rich source of polyphenolic compounds with high pro-health potential and their nutritional utility should be taken into consideration [8,9,10]. As indicated, leaves of *Rubus caesius* present strong antioxidant properties and are biologically active [5,7,8,9,10], though the plant’s antioxidant properties, chemical composition and biological activity might differ depending on the vegetation period [11,12]. During the season plants produce specific compounds essential for proper growth and keeping themself safe from environmental conditions. Many of these compounds are antioxidants and are particularly valuable in nutritional supplementation due to their cardioprotective, anti-diabetic, anti-gastrointestinal problems or anti-inflammatory properties [13]. Determination of the vegetation period when their concentration in the plant is the highest, is extremely valuable. It concerns especially compounds not widespread in nature and the synthesis of which is expensive. In such cases, for economic reasons and to reduce the cost of isolation of individual compounds, the use of plant materials rich in polyphenols in the form of extracts are sought after [11,12,13].

The aim of the study was to analyze for the first time polyphenolic composition of freshly developed *R. caesius* young leaves and stems from the first vegetation year. It was decided to examine water and ethanol extracts since they are the easiest to prepare in everyday use, as well as in traditional herbal medicine. The study has also showed analysis of antioxidant properties, as well as antihyaluronidase activity. Moreover, the antibacterial effect of leaves and stems of *Rubus caesius* was determined for the first time.

We used bacterial strains that are generally responsible for urinary tract infections, bacteremia, intra-abdominal infections and other internal infections: *Enterococcus faecalis*, *Escherichia coli*, *Salmonella enterica*, *Clostridium bifermentans* and *Clostridium sporogenes* [14,15,16,17].

## 2. Results

### 2.1. Antioxidant and Reduction Activity

The obtained extracts: leaf water extract (L-H_2_O), leaf ethanol extract (L-EtOH), stem water extract (S-H_2_O), and stem ethanol extract (S-EtOH) were tested for antioxidant activity with the use of the DPPH radical and reduction ability estimated by the FRAP test. The screening analyses of extracts showed significant antioxidant activity and reduction ability. The results given as their IC_50_ values (μg/mL), presented in Table 1, indicated significant antiradical activity of all samples (37.5–88.6 μg/mL and 38.15–87.23 μg/mL, for the DPPH and FRAP assays, respectively). Samples collected in early spring, exhibited antioxidant and reduction ability that was better than leaf water extracts collected during summer in Poland [8], although weaker than the potential of ascorbic acid. The results obtained from leaves of *R. caesius* collected during summer in Serbia, could be explained by the different climatic conditions characteristic of this country (spring leaf extracts were not tested in that research) [9]. The IC_50_ indicated the best results for both the DPPH test and ferric reducing abilities for water extracts from stems and leaves as well as stem ethanol extract (S-H_2_O > L-H_2_O > S-EtOH, Table 1). The weakest reducing and antioxidant ability was detected for ethanol extract from leaves (L-EtOH).

Antioxidant and reduction activity of extracts obtained from the leaves of European dewberry corresponds with the results obtained previously, even though they had a different harvesting time [18,19,20]. However extracts from young stems have not been analyzed.

### 2.2. The Antihyaluronidase Activity

The results of the extracts from *Rubus caesius* on the activity of the hyaluronidase enzyme revealed, that all extracts inhibited hyaluronidase in a dose–dependent manner. As shown in Table 1, the best results were revealed for the stem water (S-H_2_O) and a weaker result for the stem ethanol extract (S-EtOH). It is worth emphasizing that stem extracts were slightly less active than the standard, oleanolic acid. The obtained values were 55.24 ± 3.21 μg/mL, 68.7 ± 1.61 μg/mL and 45.71 ± 3.5 μg/mL, respectively. The leaf water extract (L-H_2_O) also presented considerable inhibition of hyaluronidase activity, resulting in IC_50_ 100.25 ± 3.73 μg/mL. Leaf ethanol extract (L-EtOH), despite increasing extract concentrations, maintained the hyaluronidase activity around 75%. According to the authors knowledge, the analysis of hyaluronidase inhibition was made in this study for the first time for extracts obtained from *Rubus caesius* leaves and stems. Studies on other species of blackberry leaves indicated a hyaluronidase inhibition (IC_50_) in the range of 127.36–180.09 μg/mL [20].

### 2.3. Antibacterial Activity

The antibacterial activity of analyzed extracts from *Rubus caesius* is listed in Table 2. For the analysis, the lyophilized extracts were dissolved in water or DMSO (), resulting in different antibacterial activities which could be related to the solubility and antioxidant ability of the plant metabolites in different solvents [21]. DMSO concentration did not influence the viability of bacterial strains; controls for antimicrobial activity for water or DMSO were also performed.

The solutions obtained by dissolving the extracts of stems and leaves in water showed the highest growth inhibitory activity against Gram-positive bacteria: *Clostridium sporogenes* and *Clostridium bifermentans* in the range of 0.5 to 0.0156 mg/mL, and *Enterococus faecalis* in the range of 1.25–0.625 mg/mL (except for the aqueous extract of the stems dissolved in water). In contrast, the inhibitory activity against Gram-negative rods was 5 mg/mL or higher. The solutions obtained by dissolving extracts in DMSO showed an inhibitory activity against anaerobic bacteria (*Clostridium*) in the range of 5–1.25 mg/mL, and for other bacteria 5 mg/mL or higher. The lowest bactericidal concentration (MBC 0.5 mg/mL) was shown by water solutions of stems extracts against Gram-positive anaerobic bacteria (*Clostridium*), *Enterococcus faecalis*, *Escherichia coli* and *Salmonella enterica*. In this case, the extract solutions showed bactericidal concentration above 0.5 mg/mL.

Antibacterial activity of young *Rubus caesius* leaves and stems, has been analyzed in our study for the first time, though blackberry leaves of other species were analyzed on *Escherichia coli*, *Salmonella enterica*, *Enterococcus faecalis strains* with good results [18,20]. Also, the influence of leaves from genus *Rubus* on Gram-positive anaerobic bacteria (*Clostridium*) was tested for the first time.

All these studies indicated that leaves of blackberry can be a good source material for antibacterial purposes.

### 2.4. Characterization of Flavonoid Glycosides and Flavonoid Aglycones Using Reversed Phase Liquid Chromatography/Electrospray Ionization Triple Quadrupole Mass Spectrometry (LC-ESI-MS/MS)

The analysis comprised qualitative and quantitative analysis of flavonoid glycosides and flavonoid aglycones in water and ethanol extracts from young vegetative shoots (leaves and stems) of *Rubus caesius*. Flavonoid profiles with quantification of the plant compounds is important for the possible use of the plant material as a nutraceutical, food or medicine. Among analyzed extracts, ten flavonoid aglycones were detected i.e., catechin, taxifolin, luteolin, eriodictyol, quercetin, apigenin, kaempferol, isokaempferide, sakuranetin, and rhamnazin, as listed in Table 3 and Figure S1.

Among them sakuranetin was not present or was below the limit of quantification in all the tested extracts. The highest amount of flavonoid aglycones was quantified in ethanol extracts of both leaves and stems. This observation can be explained with the better solubility of flavonoid aglycones in ethanol. Among others, catechin was present in the highest quantity, especially in S-EtOH, indicating the ethanol extract of the stem is a good source of this compound. A three times lower amount of catechin was detected in L-EtOH (159.0 ± 0.9 and 44.7 ± 1.0 ng/mg, respectively). This aglycone was not detected in the water extracts. Taxifolin and quercetin were quantified in all extracts, with the highest amount in L -EtOH. Luteolin and eriodictyol were quantified only in the leaf extracts, with the predominance in ethanol extracts. Apigenin and isokaempferide were detected in ethanol extracts, while kaempferol and rhamnazin were quantified only in L-EtOH extracts.

Ten flavonoid glycosides (rutin, hyperoside, luteoloside, isoquercetin, eriodictyol-7-glucopyranoside, astragalin, quercitrin, tiliroside, apigenin 7-*O*-glucoside and naringenin 7-*O*-glucoside) were detected in the analyzed extracts from *Rubus caesius* (Table 3 and Figure S2). Rutin and hyperoside were quantified in all extracts, with far higher amounts in ethanol preparations. Water extracts exhibited low amounts of flavonoid glycosides, except the previously mentioned rutin and hyperoside. The presence of isoquercetin, astragalin, quercitrin and tiliroside was detected in L-H_2_O and S-H_2_O, though their content was very low (below the limit of quantification).

Other quantified flavonoid glycosides in ethanol extracts were: isoquercetin, astragalin and naringenin-7-*O*-glucoside and tiliroside. In ethanol leaf extracts luteoloside was additionally quantified. It is worth emphasizing, that in ethanol leaf extract flavonoid glycosides were detected in higher amounts than in the ethanol stem extract. The only exception was isoquercetin, with a lower concentration in leaf ethanol extract, than in stem ethanol extract (243.67 ± 9.07 and 363.67 ± 16.44 ng/mg, respectively).

From all quantified flavonoid aglycones and flavonoid glycosides, tiliroside detected in ethanol extract from leaves presented the highest amount reaching 1573.33 ± 23.09 ng/mg.

*Rubus caesius* leaves contain ellagitannins, phenolic acids and polyphenols like derivatives of quercetin, kaempferol, luteolin and apigenin, detected by other authors, although their the quantitative content cannot be compared with each other because the harvest of leaves was in a different growing season [7,8,19,22]. Quercetin and hyperoside are widely distributed in leaves among *Rubus* L. species, with the highest amount in stone bramble *R. saxatilis* [7]. Grochowski et al. detected quercetin and kaempferol derivatives and phenolic acids, and also indicated that the lowest total polyphenolic content was in the water extract [8,10], which corresponds to our results, that ethanol extracts are richer in polyphenolic compounds.

## 3. Discussion

The European dewberry is a wild-growing shrub, cultivated also as a source of fruit rich in pro-health antioxidant compounds: anthocyanins, phenolics or flavonoids and tannins [23,24]. Leaves of *Rubus* species are used traditionally to treat diarrhea, hemorrhoids and skin problems [25], although polyphenolic profile, antimicrobial activity and hyaluronidase inhibition of extracts from young leaves and stems of *Rubus caesius* has not been fully analyzed. Our results confirm previous reports that European dewberry leaves can be used as a good source of strong antioxidants with pro-healthy properties [7,8,9,10,26]. However, the properties of antioxidant, antihyaluronidase and antibacterial activity of young stems and leaves of *Rubus caesius*, presented in this paper, are reported for the first time. Our results are significant, especially as there are no reports about chemical constituents and biological properties of stems from *Rubus caesius*.

*Enterococcus faecalis,* the Gram-positive cocci, is part of the gastrointestinal microbiota of almost all animals, including humans. Infections occurring mainly among hospitalized patients, caused by microbes of the genus *Enterococcus* (most notably *E. faecalis and E. faecium*) include urinary tract infections, bacteremia, intra-abdominal infections, and endocarditis [27]. *Rubus caesius* water and ethanol extracts from leaves exhibited strong activity against *Enterococus faecalis*, that confirms the legitimacy of the use of tea infusions from the leaves in diarrhea, gastrointestinal pain, or problems with urination. As indicated in our study, the other sensitive microbe to extracts of European dewberry (both leaves and stems) is *Clostridium*—the Gram-positive, anaerobic, spore forming bacteria. Among this group, *C. bifermentans* is a commensal in the gut, oral cavity and female genital tract and can be found in soil, sewage, feces and marine sedimentations. This organism has recently been found to be associated with septic arthritis, empyema, osteomyelitis, soft tissue infection, brain abscess, bacteremia and endocarditis; while *C. sporogenes* is known to be responsible for clostridial myonecrosis, or gas gangrene—a fast, progressive, life-threatening skeletal muscle infection [14,15]. Introduction of young green parts of European dewberry to the diet, both stem and leaves, could also limit bacterial growth of pathogenic serotypes of *Escherichia coli* and *Salmonella enterica*, that are major agents of gastroenteritis and can also cause systemic infections in animals and humans including parenteral urinary tract, blood and central nervous system infections [16,17]. The present manuscript indicates also, that analyzed bacterial strains are more sensitive to extracts dissolved in water than in DMSO. Water as a natural component of cells and solvents, in this case could be a better solvent for the plant compounds of extracts than DMSO. Only *S. enterica*, was more sensitive to the samples dissolved in DMSO, which might suggest, that this strain is sensitive to other biologically active chemicals, present in all samples and for which DMSO is a better solvent. Further analysis of antibacterial activity of extracts from *R. caesius* dissolved in water and DMSO are essential. Additionally, antibacterial activity of European dewberry can be enhanced by limiting the activity of hyaluronidase, the enzyme responsible for hyaluronan decomposition and induction of the inflammation process [28]. Stem extracts inhibited hyaluronidase slightly weaker than the standard, oleanolic acid. Also, the stem water extracts may be used in the everyday diet as an important antioxidative factor. The biological and antioxidant activity of extracts from young leaves and stems from *Rubus caesius* are connected with the rich polyphenolic profile of the extracts. FRAP and DPPH tests indicated that solutions used in the extraction process (water and ethanol) are sufficient to extract chemical constituents capable to fight oxidative stress and protect macromolecules from degradation [13]. The high antioxidant and reduction ability of extracts from young leaves and stems indicates significant chemical composition of the samples. Our research was concentrated in flavonoid aglycones and flavonoid glycosides widely distributed among *Rubus* L. species, especially in fruits. The compounds were quantified and the study indicated a positive correlation between polyphenolic content and antioxidant potency of the ethanol extracts of both leaves and stems. Significant reduction and antioxidant abilities of water extracts suggest the presence of other potent chemicals in the young leaves and stems. Among them can be polyphenolic acids or tannins detected previously in leaves collected during other vegetation periods [7,8,9,10].

It has been proven that eating food rich in polyphenolic compounds reduces the level of oxidative stress. Polyphenols have the ability to protect macromolecules, such as DNA, collagen or cell membranes, against the action of free radicals. Despite low absorption from the intestines, the consumption of natural products with high polyphenol content as a source of antioxidants is recommended [3,29]. Despite the low amount of flavonoid aglycones in the studied extracts, catechin is the exception, with a high content detected in young stems. Among flavonoid glycosides hyperoside, isoquercetin, astragalin and tiliroside (with a remarkable amount in leaves) were detected in abundant quantity, especially in the ethanol extracts. The presence of potent, biologically active flavonoids, with a widely studied spectrum of activity might have a significant influence in the nutritional properties of young leaves and stems [10,30,31].

## 4. Conclusions

Our study indicates for the first time that young leaves and stems, especially in ethanol extracts of *Rubus caesius* are valuable, polyphenolic-rich materials with biologically active rutin, tiliroside, hyperoside, and astragalin. Additionally, extracts from stems indicated a high ability to inhibit hyaluronidase activity, an inducer of the inflammation process. Antibacterial activity of all the obtained extracts, especially against *Clostridium* strains, should be emphasized. Further study on the biological activity and antibacterial properties of the selected plant fractions and compounds should be conducted and extended to other bacterial strains. The results of our research show that freshly developed leaves and stems of *Rubus caesius* can be collected and used in early spring for preparation of extracts useful in an everyday diet due to antibacterial, antioxidative, and antihyaluronidase activities and a rich source of polyphenolic compounds with proven pro-health properties.

## 5. Materials and Methods

### 5.1. Plant Material and Extracts Preparation

The first-year stems (primocanes) with freshly developed leaves of *Rubus caesius* L. were collected during the spring of 2021. Botanical identification was made by the Authors. The voucher specimen was deposited in the Herbarium of Medical University of Gdańsk, Poland (GDMA Herbarium No. 4636). The leaves (30 g) and stems (30 g) were separated, pulverized and subsequently extracted with ethanol 70% (*v*/*v*) or water (3 × 100 mL in an ultrasound bath for 15 min at room temperature(Unitra UM1, UNIMASZ OLSZTYN, Poland). Extracts were filtered and concentrated with a rotating evaporator (Büchi RotawaporR-200, Büchi Labortechnik AG, 9230 Flawil, Switzerland), frozen at −20 °C and lyophilized (Alpha 1-2 LDplus, Christ^®^, Germany).

### 5.2. Chemicals

Analytical standards of apigetrin (apigenin 7-glucoside), chrysin, prunetin, rhamnazin, morin, isoquercetin, vitexin, isovitexin, quercitrin, kaempferitrin, taxifolin, luteolin, 3-*O*-methylquercetin, kaempferol, hyperoside, rutin, isorhamnetin, isokaempferide, rhamnetin, sakuranetin, luteoloside, eriodictyol-7-*O*-glucopyranoside, narirutin, naringin, LC grade acetonitryl, DMSO, DPPH radical, ampicillin, bovine serum, ferric chloride, hyaluronidase from bovine testes type I -S, sodium phosphate buffer pH 7, oleanolic acid, 2,4,6-tris(2-pyridyl)-s-triazine TPTZ, and hyaluronic acid were obtained from Sigma-Aldrich Fine Chemicals (St. Louis, MI, USA). Naringenin, astragalin, apigenin, tiliroside, and nicotiflorin were purchased from Roth (Karlsruhe, Germany). Dihydromyricetin was supplied by LGC (LGC Group, Teddington, UK), and laricitrin by Extrasynthese (Lyon, France). Quercetin was obtained from Fluka (Buchs, Switzerland). Catechin, luteolin-7-*O*-glucoside, luteolin 3,7-diglucoside, naringenin 7-*O*-glucoside, eriodictyol, and myricetin were supplied by ChromaDex (Irvine, CA, USA). LC-MS grade water was prepared using a Millipore Direct-Q3 purification system (Bedford, MA, USA). Ethanol was obtained from Avantor Performance Materials (Gliwice, Poland).

### 5.3. The DPPH Assay

The DPPH radical scavenging assay was performed according to the previous method [32]. 100 µL of different concentrations of extracts, were mixed with 100 µL of 0.06 mM DPPH methanolic solution and incubated at room temperature in the dark for 30 min. Afterwards the absorbance was determined at 510 nm by the use of the 96-well microplate reader (Epoch, BioTek System, Winooski, VT, USA). Ascorbic acid was used as the standard.

DPPH reduction was calculated by the following equation:

DPPH reduction (%) = [Acontrol − Aextract)/Acontrol] × 100%

The antioxidant activity of the extracts was expressed as the IC_50_ value (the concentration of the analyzed extract or standard substance that causes a decrease in the non-reduced form of the DPPH radical by 50%). The IC_50_ value was calculated with the program GraFit v.7.0 (East Grinstead, West Sussex RH19 3AU, UK Erithacus Software). The assay was conducted using three replicates each. The results are presented with a standard deviation ± (SD). The statistical significance was determined with a one-way ANOVA with the post-hoc Tukey’s test (*p* < 0.05).

### 5.4. FRAP Assay

The reducing ability of analyzed *Rubus caesius* extracts were established with the FRAP test, based on the reduction of Fe^+3^ to Fe^+2^ [33]. Briefly: 30 μL of serial dilutions of the extracts and standard substance were placed in a 96-well plate and mixed with 170 μL of the freshly prepared reaction mixture (0.3 M acetate buffer: 10 mM TPTZ in 40 mM HCl: 20 mM FeCl_3_ × 6H_2_O in the ratio 10: 1: 1). The plate incubation at room temperature was for 20 min, then the absorbance was read at 593 nm. The percentage of reduced iron ions was determined from the calibration curve plotted for ascorbic acid (1–1000 µg/mL).

The reducing activity of the extracts is shown as the IC_50_ value (the concentration of the analyzed extract or standard substance that causes a decrease in the reduction of Fe^3+^ to Fe^2+^ by 50%). The IC_50_ value was calculated with the program GraFit v.7.0 (Erithacus Software). The assay was conducted using three replicates. The results are presented with a standard deviation ± (SD). The statistical significance was determined with one-way ANOVA with the post-hoc Tukey’s test (*p* < 0.05).

### 5.5. Antihyaluronidase Activity

The inhibition of hyaluronidase activity by extracts from *Rubus caesius* was evaluated spectrophotometrically by a modified method [34]. The ability to inhibit the activity was determined based on precipitation of undigested hyaluronic acid (HA) with acid albumin. Sodium phosphate buffer (50 µL, 20 mM, pH 7.0; with 77 mM NaCl and BSA 0.01%), hyaluronidase (50 µL, 100 U/mL), and 10 µL of series of dilutions of the analyzed extracts were incubated at 37 °C for 10 min. Afterwards, 50 µL of HA (0.03 % in acetate buffer, pH 5.35) was added and incubated at 37 °C for 45 min. The undigested HA was precipitated with 200 µL acid albumin solution (0.1% bovine serum albumin in 24 mM sodium acetate and 79 mM acetic acid, pH 3.75). The mixture was incubated at room temperature for 10 min, the absorbance of the reaction mixture was measured at λ = 600 nm using the 96 well microplate reader (Epoch, BioTek System, Winooski, VT, USA). Oleanolic acid was used as the standard. The absorbance in the absence of enzyme was used as the blind control. The percentage of inhibition was calculated as:

Hyaluronidase inhibition = [A_extract_ − A_control_)/A_hyaluronic acid_ − A_control_] × 100%

The antihyaluronidase activity of the extracts was expressed as the IC_50_ value (the concentration of the analyzed extract or standard substance that causes a decrease in hyaluronidase activity by 50%).

The assays were conducted three replicates each. The results are presented with a standard deviation ± (SD). The statistical significance was determined with a one-way ANOVA with the post-hoc Tukey’s test (*p* < 0.05).

### 5.6. Microorganism Species

*Enterococcus faecalis* ATCC1299 and *Escherichia coli* ATCC8739 were obtained from the ATCC collection (ATCC, Manassas, VA, USA); *Clostridium sporogenes* ATCC19404, *Clostridium bifermentans* ATCC638 and *Salmonella enterica* ATCC13076 came from Department of Pharmaceutical Microbiology of Medical University of Gdańsk collection.

Brain–heart infusion broth (BHI, Becton Dickinson, Franklin Lakes, NJ, USA) was used and supplemented with 10% bovine serum for *Clostridium sporogenes* ATCC19404, *Clostridium bifermentans* ATCC638, strains growth in GENbag anaerobe (BioMerieux, Marcy-l’Etile, France) at 37 °C for 48 h [35]. *Enterococcus faecalis* ATCC1299, *Escherichia coli* ATCC8739, *Salmonella enterica* ATCC13076, growth in Mueller-Hinton broth (MH cation-adjusted, Becton Dickinson, Franklin Lakes, NJ, USA) in an aerobic atmosphere at 37 °C for 48 h [36]. After determination of the bacterial viability BHI blood agar plates, or MH agar plates were used.

Active cultures for experiments were prepared by transferring cells from the stock cultures to tubes with adequate broth as described above. They were incubated without agitation for 24 or 48 h at 37 °C. The cultures were diluted with adequate broth to achieve an optical density corresponding to 10^6^ colony forming units per mL (CFU/mL) for bacteria species (except for *Clostridium sporogenes*, *Clostridium bifermentans*). For *Clostridium sporogenes*, *Clostridium bifermentans* inoculum was prepared from *Clostridium* colonies grown on BHI blood agar plates that had been incubated for 48 h in anaerobic conditions, final inoculum concentration of approximately 10^6^ CFU/mL [37].

The minimum inhibitory concentration (MIC) was determined by broth microdilution technique using 96-well plates. After filling each well with 100 μL of broth, dry test samples were dissolved in water or dimethyl sulfoxide (DMSO) in a final concentration about of 100 mg/mL. These solutions were diluted and added to the first well of each microtiter line. Dilution in geometric progression was done by transferring the mixture/dilution (100 μL) from the first to twelfth well. An aliquot (100 μL) was discarded from the twelfth well. The final concentration of the extracts used in the antimicrobial activity test ranged from 10 to 0.005 mg/mL and the reference ampicillin from 128 to 0.0625 μg/mL. Tests were incubated in adequate conditions described above at 37 °C for 48 (for *Clostridium* sp. for 48 h in anaerobic conditions). The end point was made by visual observation of growth. The MIC was considered as the lowest sample’s concentration that prevented visible growth. In addition, 100 μL of suspension from each well without growth was inoculated on agar plates to control for bacterial viability. After 48 h, incubation plates were checked for bacterial growth. The minimal bactericidal concentration (MBC) was defined as the minimal concentration of compounds required to kill all of the organisms [38].

### 5.7. LC-ESI(-)-MS/MS Method

The LC-MS analysis of flavonoids was conducted using a slightly modified method previously described by Łyko et al. [39]. The chromatographic separation was performed on an Eclipse XDB-C18 analytical column (4.6 × 150 mm, 5 µm; Agilent Technologies, Santa Clara, CA, USA) at 25 °C using an Agilent 1200 Series HPLC (Agilent Technologies, USA). The mobile phase consisted of 0.1% FA in water solution (A) and 0.1% FA in ACN solution (B), with the flow rate and gradient elution the same as described by Olech et al. [40]. Detection and qualification of analytes was performed on a 3200 QTRAP mass spectrometer equipped with a Turbo V™ source and an electrospray ionization (ESI) probe (Sciex, Redwood City, CA, USA) using a multiple reaction monitoring (MRM) mode. The mass spectrometer parameters were as follows: ion spray voltage at −4500 V, temperature at 500 °C, CUR gas at 23 psi, gas1 and gas2 at 50 and 60 psi. Data acquisition and processing were performed using Analyst 1.5 software (Sciex, Redwood City, CA, USA). The optimized QTRAP settings were determined experimentally for each compound and given in Table S1 in the Supplementary Material. The corresponding analytical standards were applied to generate calibration curves for detected compounds, based on the peak areas of their most intense MRM transitions. The limit of detection and limit of quantification were determined at a signal-to-noise ratio of 5:1 and 10:1, respectively (Table S2; Supplementary Material). The LC-MS analysis was conducted at least three times for each sample and standard solution.

## Figures and Tables

**Table 1:** Ferric reducing antioxidant power (FRAP, IC_50_), radical scavenging potential (DPPH, IC_50_) and hyaluronidase inhibitory activity (antihyaluronidase, IC_50_) of the extracts from *Rubus caesius* [μg/mL of the dry weight].

	Extracts of *Rubus caesius*				Standards	
	L-H_2_O ^a^	S-H_2_O ^b^	L-EtOH ^c^	S-EtOH ^d^	Ascorbic Acid	Oleanolic Acid
FRAP	47.25 ± 1.88 ^a,b^	38.15 ± 2.05 ^b,a,d^	87.23 ± 0.89	50.11 ± 1.08 ^d,b^	4.75 ± 0.6	n/a
DPPH	45.9 ± 3.9 ^a,c,d^	37.5 ± 4.7 ^b,c,d^	88.6 ± 7.4 ^c,a,b,d^	68.5 ± 5.2 ^d,a,b,c^	7.2 ± 0.7	n/a
A-H	100.25 ± 3.73 ^a,b,d^	55.24 ± 3.21 ^b,a,d^	n.r.	68.7 ± 1.61 ^d,a,b^	n/a	45.71 ± 3.5

Mean values of three replicates ± SD; n.r.: IC_50_ value was not reached in the analyzed extract concentrations; n/a: not analyzed; A-H—antihyaluronidase activity. The statistical significant differences (ANOVA, *p* < 0.05, with post-hoc Tukey’s) among the results are indicated as a,b,c,d in the table, where a is the results of L-H_2_O, b-the results of S-H_2_O, c-the results of L-EtOH, and d-the results of S-EtOH. All the results are significantly different to the control (ascorbic or oleanolic acid depending on the used method; Student’s *t*-test, *p* < 0.05).

**Table 2:** Antibacterial activity of the extracts from *Rubus caesius* [mg/mL], dissolved in water or DMSO, expressed as MIC and MBC.

	L-H_2_O DMSO		L-H_2_O Water		S-H_2_O DMSO		S-H_2_O Water		L-EtOH DMSO		L-EtOH Water		S-EtOH DMSO		S-EtOH Water		Ampicillin
Bacteria Species	MIC	MBC	MIC	MBC	MIC	MBC	MIC	MBC	MIC	MBC	MIC	MBC	MIC	MBC	MIC	MBC	MIC
*E. faecalis* ATCC51299	10	10	0.625	5	10	10	5	10	10	10	1.25	>10	10	10	1.25	10	0.0025
*E. coli* ATCC8739	10	>10	>5	>5	>10	>10	>5	>5	10	>10	>5	>5	>10	>10	>5	>5	0.0039
*S. enterica* ATCC13076	5	5	5	>10	>5	>5	10	>10	5	>5	10	>10	5	>5	10	>10	0.0005
*C. bifermentans* ATCC638	1.25	>5	0.5	>0.5	5	5	0.0625	0.5	2.5	>5	0.0156	>0.5	2.5	>5	0.0625	0.5	0.016
*C. sporogenes* ATCC19404	2.5	>5	0.03125	>0.5	5	>5	0.0156	0.5	2.5	>5	0.0156	>0.5	2.5	>5	0.0156	0.5	0.00125

*E. faecalis*—*Enterococcus faecalis*; *E. coli*—*Escherichia coli*; *S. enterica*—*Salmonella enterica*; *C. bifermentans*—*Clostridium bifermentans*; *C. sporogenes*—*Clostridium sporogenes*; MIC—minimal inhibitory concentration; MBC—minimal bactericidal concentration.

**Table 3:** Content (ng/mg of dry extract) of flavonoid aglycones and flavonoid glycosides detected in extracts from *Rubus caesius* leaves (L-H_2_O, L-EtOH) and stems (S-H_2_O, S-EtOH) using LC-ESI-MS/MS with MRM.

Flavonoid Aglycones										
*Rubus* Extract	Catechin	Taxifolin	Luteolin	Eriodictyol	Quercetin	Apigenin	Kaempferol	Isokaempferide	Sakuranetin	Rhamnazin
**L-H_2_O**	0	8.23 ± 0.41	0.30 ± 0.02	0.58 ± 0.03	12.58 ± 0.43	BQL	BQL	0	0	0
**S-H_2_O**	0	9.45 ± 0.07	0	0	2.84 ± 0.18	BQL	0	0	0	0
**L-EtOH**	44.70 ± 0.95	10.30 ± 0.17	2.79 ± 0.10	0.69 ± 0.01	26.07 ± 0.96	16.43 ± 0.40	2.37 ± 0.10	19.77 ± 0.31	BQL	2.48 ± 0.08
**S-EtOH**	**159.00 ± 1.00**	8.75 ± 0.20	BQL	BQL	17.40 ± 0.20	0.30 ± 0.01	0	15.93 ± 0.65	0	BQL
**Flavonoid Glycosides**										
***Rubus*** **Extract**	**Rutin**	**Hyperoside**	**Luteoloside**	**Isoquercetin**	**Eriodictyol-7-glucopyranoside**	**Astragalin**	**Quercitrin**	**Apigenin** **7-*O*-glucoside**	**Naringenin** **7-*O*-glucoside**	**Tiliroside**
**L-H_2_O**	38.31 ± 1.09	181.45 ± 5.64	0	BQL	0	BQL	BQL	0	0	BQL
**S-H_2_O**	16.20 ± 0.60	101.50 ± 1.32	0	BQL	0	BQL	BQL	0	0	BQL
**L-EtOH**	115.60 ± 9.15	**1066.67 ± 32.15**	32.30 ± 1.11	243.67 ± 9.07	BQL	342.00 ± 7.55	BQL	BQL	52.93 ± 2.06	**1573.33 ± 23.09**
**S-EtOH**	140.67 ± 2.52	**1096.67 ± 35.12**	BQL	363.67 ± 16.44	BQL	99.33 ± 1.65	BQL	BQL	24.07 ± 1.50	408.00 ± 12.00

The results are presented as mean values of three replications ± SD. BQL—compound detected, but its concentration was below the quantification limit.

## Data Availability

All data are included in the manuscript.

## Supplementary Materials

The following supporting information can be downloaded at: https://www.mdpi.com/article/10.3390/molecules27196181/s1, Figure S1: LC-MS chromatograms obtained in multiple reaction monitoring (MRM) mode of flavonoid aglycones detected in sample *Rubus* L-EtOH; 1—Catechin; 2—Taxifolin; 3—Luteolin; 4—Eriodictyol; 5—Quercetin; 6—Apigenin; 7—Kaempferol; 8—Isokaempferide; 9—Sakuranetin; 10—Rhamnazin.; Figure S2: LC-MS chromatograms obtained in multiple reaction monitoring (MRM) mode of flavonoid glycosides detected in *Rubus* L-EtOH. 1—Rutin; 2—Hyperoside & Isoquercetin; 3—Luteoloside; 4—Eriodictyol-7-glucopyranoside; 5—Astragalin; 6—Quercitrin; 7—Apigenin 7-O-glucoside; 8—Naringenin 7-O-glucoside; 9—Tiliroside.; Table S1: Summary of optimized QTRAP parameters for the LC-MS analysis of flavonoids. Abbreviations: Q1/Q3—m/z values for precursor and fragment ion detected in Q1 and Q3 quadrupole, respectively (tracked MRM transitions); declustering potential (DP); entrance potential (EP); collision cell exit potential (CXP); collision energy (CE); collision cell exit potential (CXP).; Table S2: Analytical parameters used for quantitative determination of flavonoids detected in samples.

## Sample Availability

Samples of the compounds are available from the authors.
