# Supplementary material for: Polyphenolic Characterization, Antioxidant, Antihyaluronidase and Antimicrobial Activity of Young Leaves and Stem Extracts from Rubus caesius L
Source: Molecules. 2022 Sep 21;27(19):6181. doi: 10.3390/molecules27196181 (PMC9572312; doi:10.3390/molecules27196181)

Table S1. Summary of optimized QTRAP parameters for the LC-MS analysis of flavonoids. Abbreviations: Q1/Q3 – m/z values for precursor and fragment ion detected in Q1 and Q3 quadrupole, respectively (tracked MRM transitions); declustering potential (DP); entrance potential (EP); collision cell exit potential (CXP); collision energy (CE); collision cell exit potential (CXP).

Compound	Retention time [min]	Q1/Q3 [m/z]	DP [V]	EP [V]	CEP [V]	CE [eV]	CXP [V]
		**Flavonoid aglycones**					
Catechin	9.64	288.8/244.9	-45	-4.5	-16	-16	-4
		288.8/109	-45	-4.5	-16	-32	0
Epigallocatechin gallate	11.20	457/169.1	-25	-10	-28.6	-30	-3
		457/125	-25	-10	-28.6	-30	-3
Dihydromyricetin (Ampelopsin; Ampeloptin)	12.10	319/193	-25	-10	-23.5	-30	-3
		319/125	-25	10	-23.5	-30	-3
Naringenin	14.52	270.8/119	-50	-11.5	-12	-34	0
		270.8/150.9	-50	-11.5	-12	-22	0
Taxifolin	15.17	302.7/124.9	-45	-3.5	-18	-26	0
		302.7/284.8	-45	-3.5	-18	-14	-4
Myricetin	16.57	316.7/136.9	-55	-9	-14	-32	0
		316.7/150.9	-55	-9	-14	-26	0
Morin	17.48	300.7/124.9	-50	-3.5	-20	-24	0
		300.7/106.9	-50	-3.5	-20	-30	0
Luteolin	17.84	284.7/132.9	-75	-9	-18	-38	0
		284.7/150.9	-75	-9	-18	-26	0
Eriodictiol	17.90	286.7/134.9	-45	-6	-12	-32	0
		286.7/150.9	-45	-6	-12	-18	-2
Laricitrin (3'-O-Methylmyricetin)	17.92	330.97/151	-25	-10	-23.9	-30	-3
		330.97/315.9	-25	10	-23.9	-30	10
Quercetin	17.94	300.7/150.9	-60	-2.5	-12	-26	0
		300.7/178.8	-60	-2.5	-12	-20	-2
3-*O*-Methylquercetin	18.11	314.7/299.8	-55	-9.5	-22	-18	-4
		314.7/270.8	-55	-9.5	-22	-26	-4
Apigenin	18.67	268.8/117	-70	-9.5	-12	-44	0
		268.8/106.8	-70	-9.5	-12	-34	0
Kaempferol	18.87	284.7/116.8	-70	-5	-12	-46	0
		284.7/93	-70	-5	-12	-52	0
Isorhamnetin	18.99	314.7/299.7	-65	-2.5	-26	-20	-4
		314.7/150.9	-65	-2.5	-26	-30	0
Isokaempferide	19.18	298.8/283.9	-50	-4.5	-12	-18	-4
		298.8/226.9	-50	-4.5	-12	-28	-2
Rhamnetin	20.10	314.7/165	-60	-5.5	-18	-24	0
		314.7/120.9	-60	-5.5	-18	-36	0
Sakuranetin	21.70	284.7/118.9	-60	-5.5	-12	-34	0
		284.7/164.8	-60	-5.5	-12	-20	-2
Chrysin	21.82	252.8/208.9	-80	-10	-14	-22	-2
		252.8/142.9	-80	-10	-14	-26	0
Prunetin	21.98	282.8/267.7	-55	-12	-18	-20	-4
		282.8/238.7	-55	-12	-18	-26	-2

Rhamnazin	22.44	328.7/270.8<br>328.7/313.8	-70<br>-70	-3<br>-3	-28<br>-28	-26<br>-14	-2<br>-4
		**Flavonoid glycosides**					
Kaempferol 3-rutinoside 4'-glucoside	10.40	754.8/592.8<br>754.8/284.6	-105<br>-105	-3.5<br>-3.5	-20<br>-20	-60<br>-30	-10<br>-22
Kaempferol-3-*O*-galactoside-rhamnoside-7-*O*-rhamnoside (Robinin)	10.80<br>11.07	738.8/592.8<br>738.8/254.8	-90<br>-90	-4.5<br>-4.5	-24<br>-24	-36<br>-80	-24<br>-4
Kaempferol 3-glucoside-7-rhamnoside	11.25	592.7/284.9<br>592.7/430.3	-145<br>-145	-4.5<br>-4.5	-30<br>-30	-42<br>-36	-4<br>-16
Luteolin 3',7'-diglucoside	11.28	609.1/285<br>609.1/447	-70<br>-70	-7.5<br>-7.5	-28<br>-28	-50<br>-32	-4<br>-18
Quercetin 3,7-dirhamnoside	11.35	592.8/445.7<br>592.8/298.9	-90<br>-90	-4<br>-4	-26<br>-26	-48<br>-34	-4<br>-18
Quercetin-3-*O*-rutinoside (Rutin)	12.05	608.7/299.6<br>608.7/270.9	-90<br>-90	-8<br>-8	-30<br>-30	-46<br>-60	-4<br>-4
Kempferol 3,7-dirhamnoside (Kaempferitrin)	12.16	576.8/284.8<br>576.8/430.9	-80<br>-80	-4.5<br>-4.5	-28<br>-28	-42<br>-30	-4<br>-18
Apigenin – 6-*C*-glucoside (Isovitexin)	12.38	430.8/310.9<br>430.8/340.9	-65<br>-65	-4.5<br>-4.5	-18<br>-18	-28<br>-26	-4<br>-14
Apigenin – 8-*C*-glucoside (Vitexin)	12.40	430.8/310.9<br>430.8/340.9	-75<br>-75	-4.5<br>-4.5	-20<br>-20	-26<br>-34	-4<br>-14
Luteolin-7-*O*-glucoside (Luteoloside)	12.96	446.8/284.8<br>446.8/132.9	-70<br>-70	-10.5<br>-10.5	-20<br>-20	-30<br>-78	-4<br>0
Quercetin-3-*O*-galactoside (Hyperoside)	12.97	462.7/299.7<br>462.7/254.7	-70<br>-70	-4<br>-4	-18<br>-18	-28<br>-42	-4<br>-2
Quercetin-3-*O*-glucoside (Isoquercetin)	13.09	462.7/299.7<br>462.7/270.7	-85<br>-85	-1.5<br>-1.5	-20<br>-20	-30<br>-44	-4<br>-4
Eriodictyol-7-*O*-glucopyranoside	13.07	448.8/286.9<br>448.8/134.9	-75<br>-75	-4.5<br>-4.5	-20<br>-20	-24<br>-48	-4<br>-2
Kaempferol – 3-*O*-rutinoside (Nicotiflorin)	13.31	592.7/284.8<br>592.7/226.7	-65<br>-65	-12<br>-12	-30<br>-30	-38<br>-68	-2<br>-2
Naringenin-7-*O*-rutinoside (Narirutin)	13.80	578.9/270.8<br>578.9/118.9	-90<br>-90	-4.5<br>-4.5	-24<br>-24	-34<br>-76	-4<br>0
Naringenin-7-*O*-rhamnosidoglucoside	14.50	579.1/151<br>579.1/271	-80<br>-80	-4<br>-4	-26<br>-26	-54<br>-42	-2<br>-4
Kaempferol – 3-*O*-glucoside (Astragalin)	14.70	446.7/226.8<br>446.7/254.8	-75<br>-75	-9<br>-9	-20<br>-20	-54<br>-40	-2<br>-2
Quercetin 3-*O*-rhamnoside (Quercitrin)	14.87	446.7/299.7<br>446.7/270.7	-65<br>-65	-9<br>-9	-18<br>-18	-30<br>-40	-4<br>-4
Apigenin 7-*O*-glucoside (Apigetrin, Cosmosiin)	14.93	430.7/267.7<br>430.7/116.9	-70<br>-70	-9<br>-9	-20<br>-20	-38<br>-84	-4<br>0
Naringenin 7-*O*-glucoside	15.12	432.7/270.8<br>432.7/118.9	-40<br>-40	-8.5<br>-8.5	-20<br>-20	-22<br>-64	-4<br>0
Tiliroside	17.40	592.8/284.8<br>592.8/254.7	-70<br>-70	-7.5<br>-7.5	-24<br>-24	-38<br>-56	-4<br>-2

Table S2. Analytical parameters used for quantitative determination of flavonoids detected in samples.

Compound	LOD [ng/mL]	LOQ [ng/mL]	R^2	Linearity range [ng/mL]
**Flavonoid aglycones**				
Catechin	250	330	0.9982	330-6600
Taxifolin	10	20	0.9981	40-10000
Luteolin	6	16	0.9974	33-1650
Eriodictyol	33	66	0.9984	66-6600
Quercetin	66	132	0.9977	132-6600
Apigenin	15	22	0.9979	89-4470
Kaempferol	33	66	0.9977	165-3300
Isokaempferide	65	140	0.9989	140-2800
Sakuranetin	70	135	0.9990	135-2700
Rhamnazin	170	280	0.9988	280-4200
**Flavonoid glycosides**				
Rutin	120	280	0.9983	450-9000
Hyperoside	175	300	0.9983	500-7500
Isoquercetin	175	300	0.9988	250-5000
Luteoloside	50	100	0.9980	200-8000
Eriodictyol-7-glucopyranoside	100	250	0.9990	750-15000
Astragalin	100	250	0.9988	500-7500
Quercitrin	50	100	0.9986	1000-25000
Apigenin 7-O-glucoside	100	220	0.9987	500-7000
Naringenin 7-*O*-glucoside	100	167	0.9987	250-25000
Tiliroside	100	250	0.9986	750-25000

Figure S1. LC-MS chromatograms obtained in multiple reaction monitoring (MRM) mode of flavonoid aglycones detected in sample *Rubus* L-EtOH; 1 – Catechin; 2 – Taxifolin; 3 – Luteolin; 4 – Eriodictyol; 5 – Quercetin; 6 – Apigenin; 7 – Kaempferol; 8 – Isokaempferide; 9 – Sakuranetin; 10 – Rhamnazin.

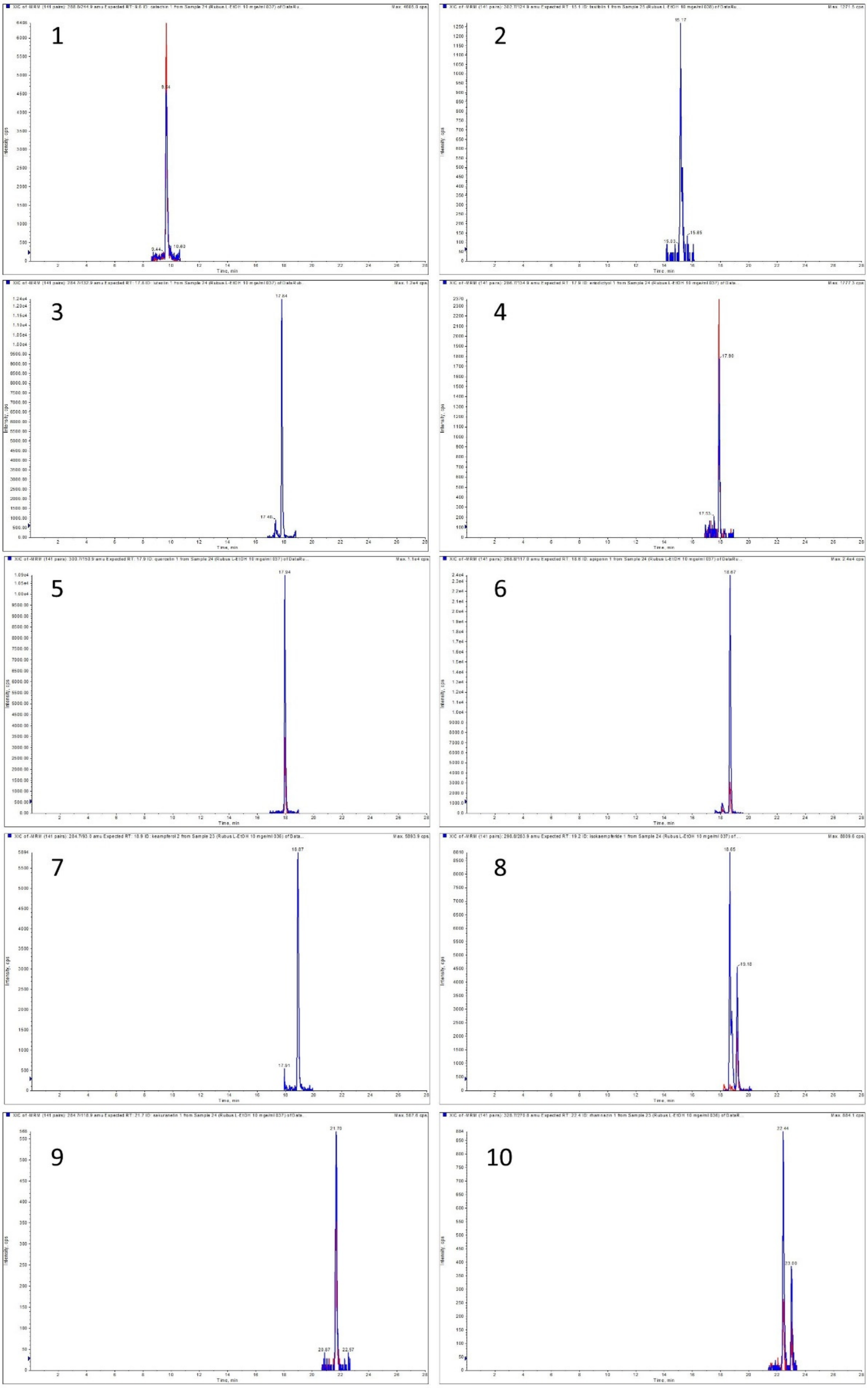

Figure S2. LC-MS chromatograms obtained in multiple reaction monitoring (MRM) mode of flavonoid glycosides detected in *Rubus* L-EtOH. 1 – Rutin; 2 – Hyperoside & Isoquercetin; 3 – Luteoloside; 4 - Eriodictyol-7-glucopyranoside; 5 - Astragalin -; 6 – Quercitrin; 7 - Apigenin 7-O-glucoside; 8 - Naringenin 7-O-glucoside; 9 – Tiliroside.

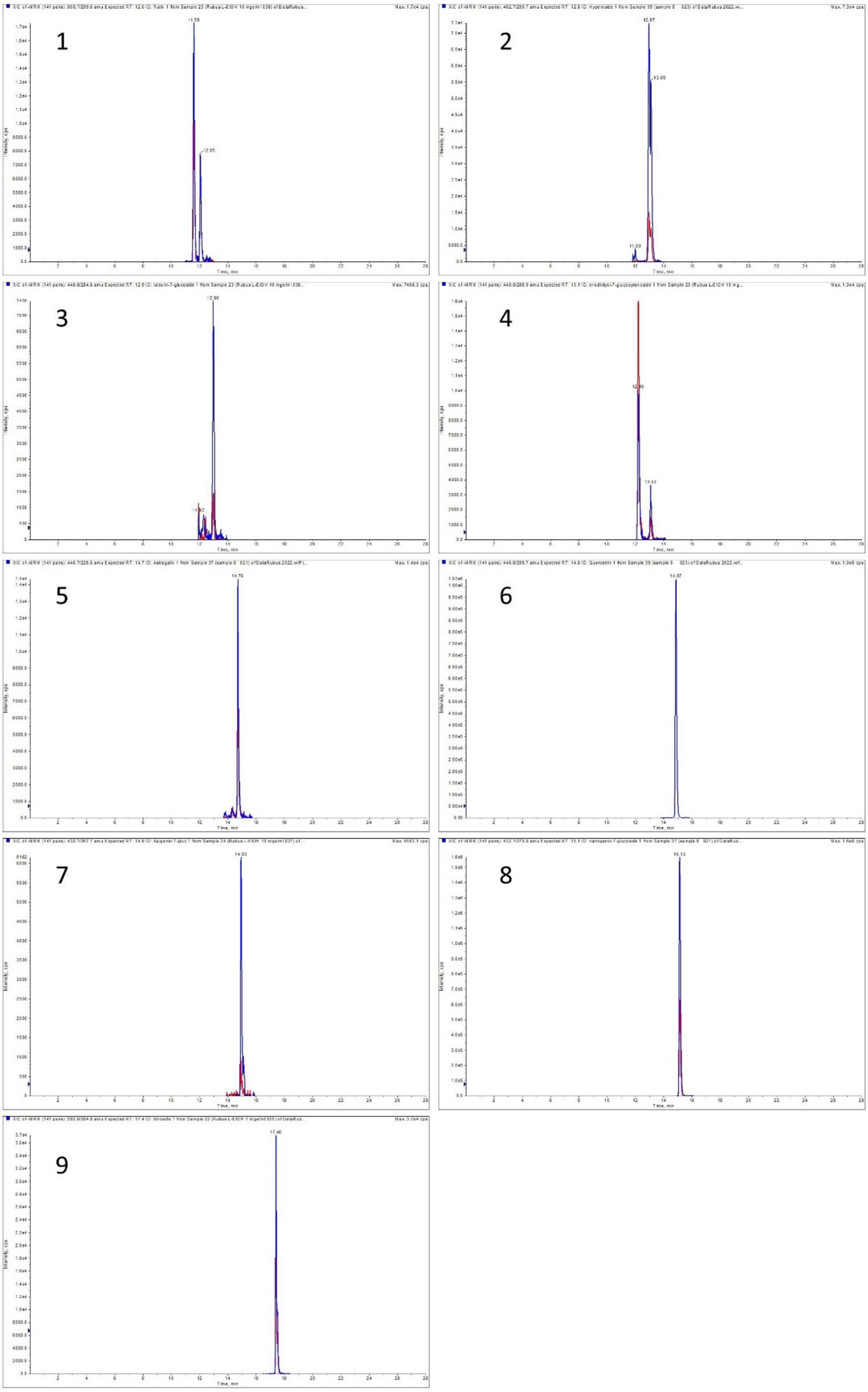

Supplement: Supplementary file 1 [file molecules-27-06181-s001.zip › molecules-1916817-supplementary.pdf]
